# Intravenous immunoglobulin and febrile status epilepticus in children with Dravet syndrome: A retrospective multicentre study

**DOI:** 10.1111/dmcn.70077

**Published:** 2025-11-12

**Authors:** Romane Marc, Nicole Chemaly, Mathieu Kuchenbuch, Caroline Espil‐Taris, Elodie Lametery, Hélène Maurey, Sylviane Peudenier, Sylvie Nguyen The Tich, Rima Nabbout, Claire Bar

**Affiliations:** ^1^ Department of Paediatric Neurology Reference Centre for Rare Epilepsies, CHU Bordeaux Bordeaux France; ^2^ Department of Paediatric Neurology Reference Centre for Rare Epilepsies, Enfants Malades University Hospital, AP‐HP, Université Paris Cité Paris France; ^3^ Université de Lorraine CHRU‐Nancy, Service de Pédiatrie, Reference Centre for Rare Epilepsies Nancy France; ^4^ Université de Lorraine, IMoPA Nancy France; ^5^ Department of Paediatric Neurology Grenoble University Hospital Grenoble France; ^6^ Department of Paediatric Neurology Competence Centre for Rare Epilepsies, CHU Bicêtre, Le Kremlin Bicêtre France; ^7^ Paediatrics Department CHU Brest, Centre de Référence Déficience Intellectuelle & Polyhandicap de Causes Rares Brest France; ^8^ CHU Lille, Reference Centre for Rare Epilepsies Lille France; ^9^ Institut Imagine INSERM UMR 1163, Translational Research on Neurological Diseases, Université Paris Cité Paris France; ^10^ CNRS, INCIA, UMR 5287, NRGen Team, University of Bordeaux Bordeaux France

## Abstract

**Aim:**

To assess the efficacy and tolerability of intravenous immunoglobulin (IVIG) in reducing febrile status epilepticus in children with Dravet syndrome.

**Method:**

We conducted a retrospective multicentre study across seven French university hospitals (2005–2022). Children with genetically confirmed Dravet syndrome who received sequential IVIG were included. Clinical data were collected over two 6‐month periods: before and after IVIG initiation.

**Results:**

Fourteen individuals (six males, eight females) were included. At IVIG initiation, all were in the stormy phase, aged 10 to 92 months, and receiving a median of four antiseizure medications. IVIG was administered every 1 to 6 weeks (0.3–0.5 g/kg per infusion). Hospitalizations for status epilepticus significantly decreased, from a median of 4 (range 0–16) at baseline to 1 (range 0–6) after treatment (*p* = 0.002). Twelve individuals improved, two remained stable. Adverse events occurred in 6 out of 14 individuals, including infusion‐related fever or seizures. Central venous access was required in six cases. IVIG was continued beyond 6 months in 11 out of 14 individuals.

**Interpretation:**

These series suggest a potential benefit of IVIG in reducing status epilepticus in selected children with Dravet syndrome. However, tolerability and feasibility issues were identified. A prospective controlled trial is warranted to further define the role of IVIG in this population.

## Graphical abstract

This multicenter retrospective study evaluated the use of intravenous immunoglobulin (IVIG) in 14 children with Dravet syndrome as a preventive strategy against febrile status epilepticus. Over a 6‐month period, IVIG was associated with a significant reduction in hospitalizations for febrile status epilepticus. These findings support its potential utility as an adjunctive treatment in selected cases.

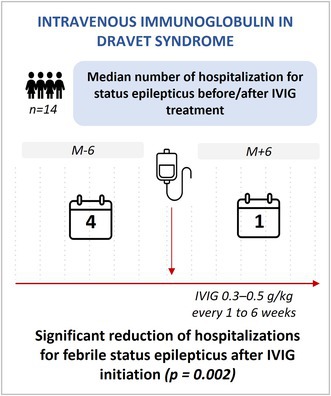

**Abbreviation**
IVIG: intravenous immunoglobulin

**What this paper adds**

- Intravenous immunoglobulin (IVIG) was used to prevent febrile status epilepticus in Dravet syndrome.
- Hospitalizations for febrile status epilepticus decreased after IVIG initiation.
- Infusion‐related adverse events were reported in nearly half of treated individuals.

Dravet syndrome is a severe developmental and epileptic encephalopathy of early childhood, defined by the International League Against Epilepsy as a genetic developmental and epileptic encephalopathy characterized by fever‐sensitive, prolonged seizures beginning in infancy, and association with subsequent developmental slowing and intractable epilepsy.^1^ Seizures typically begin within the first year of life in previously typically developing infants, often as prolonged hemiclonic or generalized tonic–clonic seizures triggered by fever.^1^, ^2^ During early childhood, nearly every febrile episode, most often due to common viral infections, or routine vaccinations can provoke status epilepticus, leading to frequent emergency department visits and intensive care admissions.^2^, ^3^ These early prolonged seizures may contribute significantly to long‐term neurological impairments.^4^ The burden extends beyond clinical aspects to psychosocial and economic spheres, as repeated hospitalizations strain family quality of life and health care system resources.^5^, ^6^

Recent guidelines recommend a stepwise therapeutic approach in Dravet syndrome.^3^, ^7^, ^8^ Initial treatments include the broad‐spectrum antiseizure medications valproate and clobazam, but these are generally insufficient to control seizures. Adjunctive therapies have since been approved specifically for Dravet syndrome: stiripentol in 2007 (EU) and 2018 (USA), cannabidiol in 2018/2019, and fenfluramine in 2020. Among these, only stiripentol has demonstrated efficacy in preventing prolonged seizures and status epilepticus.^9^ Therefore, despite current antiseizure medication, many individuals continue to experience frequent fever‐induced seizures during early childhood, prompting the consideration of additional preventive strategies. In particular, since viral infections are a major trigger for prolonged seizures and status epilepticus in young children with Dravet syndrome, one hypothesis is that reducing the frequency of febrile episodes and the associated inflammatory responses could help to decrease the incidence of such severe events. In this context, intravenous immunoglobulins (IVIG) have emerged as a potential option, owing to their dual role in infection prophylaxis and immune modulation. IVIG are purified preparations of human immunoglobulin G, obtained from pooled plasma of thousands of healthy donors. They contain a broad spectrum of antibodies and are widely used both for infection prophylaxis in immunocompromised individuals, such as those with primary antibody deficiencies or haematological malignancies, and as immunomodulatory agents in autoimmune and inflammatory diseases.^10^, ^11^, ^12^ Their potential anti‐inflammatory and neuroprotective effects have been explored in various epilepsy contexts, particularly status epilepticus and drug‐resistant childhood epilepsy syndromes such as West syndrome, Lennox–Gastaut syndrome, or Landau–Kleffner syndrome.^13^, ^14^, ^15^, ^16^, ^17^, ^18^ While some studies have suggested possible benefits, the lack of robust and consistent evidence does not currently allow for clear recommendations regarding their use.^19^ To date, no published data have specifically evaluated IVIG in individuals with Dravet syndrome. Nonetheless, sequential IVIG therapy has been empirically introduced into clinical practice. In France and Europe, a reduction in the frequency of prolonged febrile seizures was observed about a decade ago in a few immunocompetent individuals with Dravet syndrome treated with sequential courses of IVIG. Since then, several other individuals have received these IVIG treatments, with the aim of reducing the frequency of febrile infections and thus fever‐induced and prolonged seizures. However, the benefits and risks of this practice have not been formally assessed.

This study aims to report the French experience of sequential IVIG therapy in young children with Dravet syndrome, evaluating its potential to reduce febrile status epilepticus by decreasing the frequency of infections, and assessing treatment tolerance and feasibility.

## METHOD

### Participants

Patients were retrospectively identified through a survey conducted among clinicians from paediatric neurology departments of university hospitals in France within the network of the Reference Centre for Rare Epilepsies (Angers, Bordeaux, Brest, Grenoble, Lille, Paris‐Bicêtre, Paris‐Necker) from January 2005 to July 2022. Individuals were included if they had a confirmed diagnosis of Dravet syndrome, initiated IVIG treatment before the age of 18 years, had received at least three IVIG infusions, and had available clinical data for at least 6 months before and 6 months after IVIG initiation. We selected a 6‐month posttreatment evaluation to ensure adequate follow‐up for assessing the frequency of prolonged febrile seizures and status epilepticus, and to allow direct comparison with the 6‐month pretreatment baseline. IVIG was expected to exert a relatively rapid effect in case of efficacy, particularly through the prevention of infection‐related triggers. Given the average half‐life of IVIG, approximately 3 to 4 weeks, we arbitrarily excluded individuals who received fewer than three infusions. This threshold was chosen to ensure a minimal duration of exposure (around 2 months), allowing sufficient time for a potential therapeutic effect to manifest and be meaningfully evaluated against baseline seizure frequency.

### Data collection

Clinical data was collected through a standardized survey completed by clinicians following the individuals. Data collection was divided into two distinct periods: a 6‐month baseline period before IVIG initiation and a 6‐month follow‐up period after treatment initiation. For each period, information was recorded on the number of hospitalizations for status epilepticus and changes in antiseizure medications. Status epilepticus was defined according to the 2015 International League Against Epilepsy operational definition as a generalized seizure lasting longer than 5 minutes.^20^ Overall seizure frequency was not collected, as it could not be consistently retrieved from retrospective medical records and was often difficult to distinguish between febrile and afebrile events. Information regarding IVIG treatment, including dosage, infusion frequency, and duration, was also collected. Additionally, tolerance data including adverse events such as infusion‐related fever, seizures during or after infusion, and difficulties encountered during venous access were documented. The study protocol was approved by the Research Ethics Committee of Bordeaux University Hospital and conducted in accordance with the Strengthening the Reporting of Observational Studies in Epidemiology (STROBE) guidelines. Orally informed consent for both participation and publication of anonymized clinical data was obtained from all individuals or their legal guardians, in compliance with the French MR‐004 reference methodology for retrospective studies.

### Statistical analysis

Descriptive statistics are presented as medians (interquartile ranges [IQR]; range) and counts (percentages). Statistical comparisons were performed using non‐parametric tests because of the small sample size and non‐normal distribution of the data. The Wilcoxon signed‐rank test was used to compare the number of hospitalizations for status epilepticus before and after IVIG treatment. Missing laboratory values for baseline serum immunoglobulin levels were not imputed, as these data were descriptive only. Two‐tailed *p*‐values lower than 0.05 were considered statistically significant.

## RESULTS

### Population

Fourteen individuals (six males, eight females) were included in this study (Table 1). Thirteen individuals had a pathogenic *SCN1A* variant, and one had an *SCN1B* variant. The median age at seizure onset was 5 months (IQR 3.6–6 months, range 2–9 months). All individuals had drug‐resistant epilepsy and received a median of four antiseizure medications at the time of IVIG initiation (IQR 3–4, range 2–5). Eleven individuals were receiving at least the combination of valproate, stiripentol, and clobazam at inclusion. Baseline serum immunoglobulin levels were available for 9 out of 14 individuals. All values were within normal ranges, except for patient 13 who had an isolated immunoglobin G1 subclass deficiency (2.3 mg/mL; normal >4 mg/mL), while total immunoglobin G, immunoglobin A, and immunoglobin M levels remained within the normal range.

**Table 1:** Clinical characteristics, intravenous polyvalent immunoglobulin therapy, and infusion‐related events in 14 children with Dravet syndrome.

ID	Sex	Gene	Age at first seizure (months)	ASM before IGIV	Intravenous polyvalent immunoglobulin				
					Age at initiation (month)	Infusion interval	Dose	Adverse events	Venous access difficulties
1	M	*SCN1A*	9	VPA, STP, CLB	18	Monthly	0.5 g/kg	Fever‐induced seizures	CVA required
2	F	*SCN1A*	5	VPA, STP, CLB	11	Every 6 weeks	0.3–0.5 g/kg	None	CVA required
3	F	*SCN1A*	5	VPA, STP, CLB	18	Every 6 weeks	0.3 g/kg	None	CVA required
4	F	*SCN1A*	6	VPA, STP, CLB, TPM	48	Every 6 weeks	0.5 g/kg	None	None
5	F	*SCN1A*	3	VPA, STP, CLB, TPM	14	Monthly	0.3–1 g/kg	Fever‐induced seizures	CVA required
6	M	*SCN1A*	2	VPA, CLB, STP, KD	26	Monthly	0.5 g/kg	None	None
7	M	*SCN1A*	6	VPA, CLB, CBD, STP	32	Monthly	0.2–0.5 g/kg	Fever‐induced seizures	None
8	F	*SCN1A*	7	CLB, LEV, TPM	30	Monthly	0.3–0.5 g/kg	None	None
9	F	*SCN1A*	3.5	VPA, FFA	16	Monthly	0.4 g/kg	Fever‐induced seizures	None
10	F	*SCN1A*	4	KD, VPA, CLB, STP, TPM	13	Monthly	0.4 g/kg	None	Switch to subcutaneous infusions
11	F	*SCN1A*	5	VPA, STP, CLB, TPM	60	Monthly	1 g/kg	Fever without seizures	CVA required
12	M	*SCN1B*	4	VPA, STP, CLB, CBD, KD	92	Weekly	0.5 g/kg	None	Switch to subcutaneous infusions
13	M	*SCN1A*	9	VPA, TPM, CLB	10	Every 3 weeks	0.4 g/kg	None	CVA required
14	M	*SCN1A*	2.5	VPA, CLB, STP, TPM	24	Monthly	0.4 g/kg	None	None

Abbreviations: ASM, antiseizure medication; CBD, cannabidiol; CBZ, carbamazepine; CLB, clobazam; CVA, central venous access; FFL, fenfluramide; IGIV, intravenous polyvalent immunoglobulin; KD, ketogenic diet; STP, stiripentol; TPM, topiramate; VPA, valproic acid.

### IVIG therapy

IVIG were administered every 1 to 6 weeks, according to empirically defined protocols that varied among centres and were often adjusted seasonally—typically with shorter intervals in winter and longer ones in summer. During the 6‐month evaluation period, individuals received a median of six IVIG infusions (IQR 4.5–6, range 3–20). Each infusion was administered at a dose of 0.3 g/kg to 0.5 g/kg over approximately 3 hours. The choice of immunoglobulin product was based on local availability, and no significant differences in tolerance were observed between brands. IVIG treatment started at a median age of 21 months (IQR 14.5–31.5 months, range 10–92 months). Eleven of 14 individuals continued IVIG infusions beyond the initial 6‐month follow‐up. Among them, treatment was maintained on a similar schedule (mostly every 4–6 weeks), with summer interruptions reported in six cases. In five individuals, IVIG was discontinued after one or two winters following a decrease in fever‐sensitive seizures.

### Hospitalizations for status epilepticus before and after IVIG

**Figure 1:**
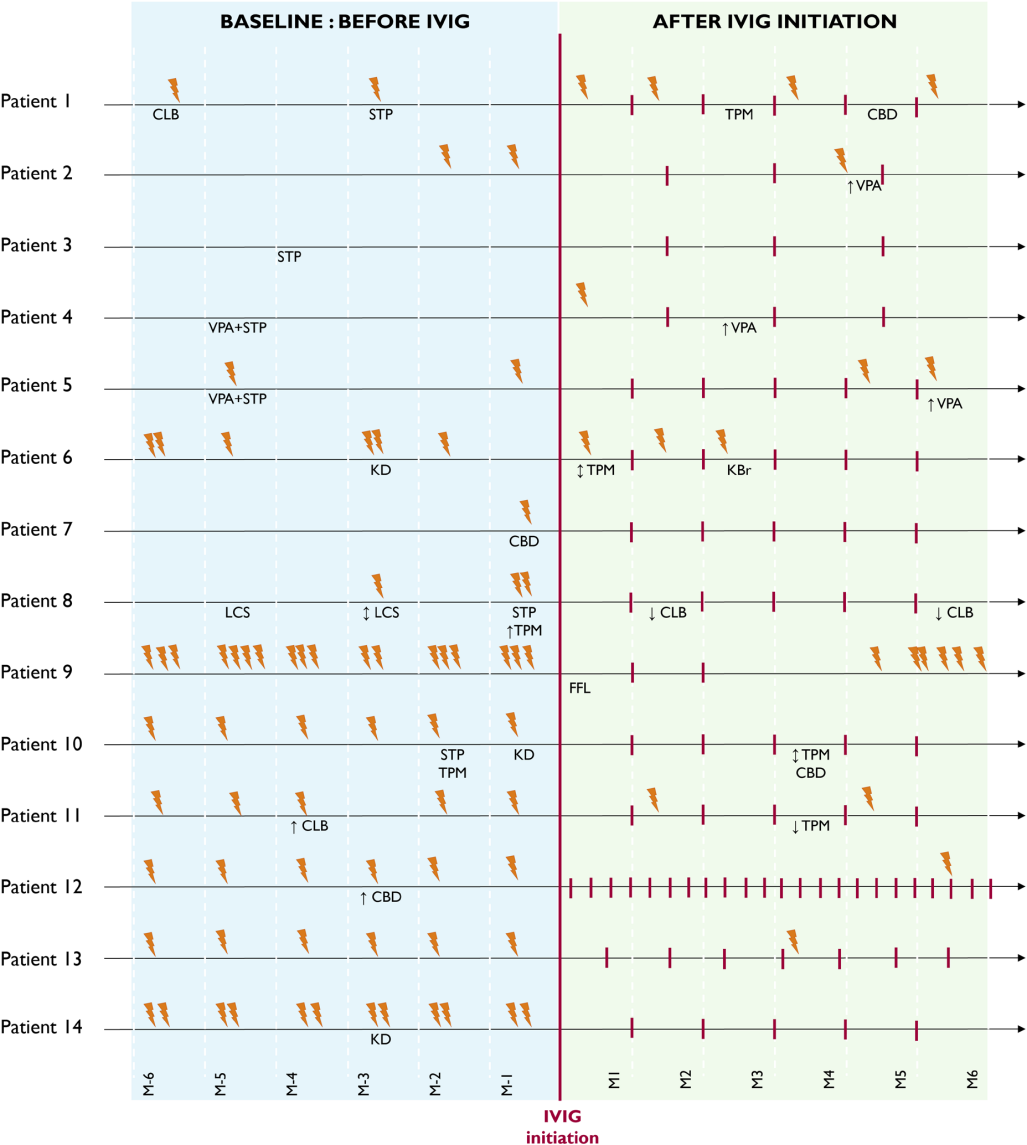
Number of hospitalizations for status epilepticus before and after initiation of intravenous immunoglobulin (IVIG) in children with Dravet syndrome. The baseline period (6 months before IVIG initiation) is shown in blue, and the IVIG treatment period (6 months following initiation) is shown in green. Each lightning bolt represents a hospitalization for status epilepticus. Red vertical bars indicate the timing of IVIG infusions. Concomitant changes in antiseizure medications are indicated below each timeline. Abbreviations: CBD, cannabidiol; CBZ, carbamazepine; CLB, clobazam; FFL, fenfluramine; IVIG, intravenous immunoglobulin; KBr, potassium bromide; KD, ketogenic diet; LCS, lacosamide; STP, stiripentol; TPM, topiramate; VPA, valproic acid.

The median number of emergency hospitalizations for status epilepticus significantly decreased from four (IQR 2–6, range 0–16) during the 6‐month baseline period to one (IQR 0–2, range 0–6) during the 6‐month IVIG treatment period (*p* = 0.002), with a median paired difference of −2 hospitalizations (IQR −4 to −1). Twelve of 14 individuals experienced a reduction in hospitalizations for status epilepticus, while two remained stable and no patient worsened. One of the stable individuals (patient 3) did not experience any hospitalization for status epilepticus during the study period. IVIG was initiated in this case because of frequent clusters of generalized tonic–clonic seizures triggered by viral upper respiratory infections. The treatment was reported as having limited clinical efficacy and was discontinued after one winter season. Individual patient trajectories, including seizure‐related hospitalizations, timing of IVIG infusions, and concomitant therapeutic adjustments, are illustrated in Figure 1.

### Treatment tolerability

Adverse events were reported in 6 out of 14 individuals. These included fever‐induced seizures during infusion in five individuals, isolated fever without seizures in one patient, and seizures without fever in two individuals. These adverse events led to the interruption of ongoing IVIG infusions in five individuals: in four cases because of fever and seizures (individuals 1, 3, 5, and 9), and in three cases because of failed venous access (individuals 1, 2, and 3). Among these, only one patient (patient 9) permanently discontinued IVIG treatment after three infusions because of recurrent febrile seizures during infusion and parental concern. Thirteen individuals initially received IVIG via the intravenous route. Among them, two were later transitioned to subcutaneous administration due to venous access difficulties. One patient received subcutaneous infusions from the outset for the same reason. In total, 6 out of 14 individuals required the placement of a central venous catheter during treatment.

## DISCUSSION

This is the first study specifically investigating the use of IVIG for the prevention of febrile status epilepticus in paediatric individuals with Dravet syndrome. Although early escalation to bitherapy or tritherapy is recommended,^3^, ^7^, ^8^ prolonged febrile seizures and status epilepticus remain frequent and difficult to manage during infancy and early childhood. In this period, nearly every infectious or postvaccination fever episode can trigger a seizure, often evolving into status epilepticus. Non‐specific preventive strategies, such as systematic antipyretic use, close fever monitoring, or ear, nose, and throat surgeries aimed at reducing recurrent infections (e.g. adenoidectomy or tonsillectomy), are frequently used in clinical practice but lack robust evidence supporting their efficacy. The persistence of febrile status epilepticus despite appropriate antiseizure therapy continues to impose a major burden on families and health care systems and may contribute to long‐term developmental impairment.^5^, ^6^, ^21^ In this context, our results suggest a possible benefit of IVIG, with a significant reduction in hospitalizations for status epilepticus following treatment initiation. Although the overall response was heterogeneous, some individuals experienced a marked and rapid improvement, suggesting a potential therapeutic effect in selected cases.

The use of IVIG in Dravet syndrome is not supported by current guidelines but has been empirically introduced in some centres. It is primarily based on the hypothesis that enhancing humoral immunity may help reduce the frequency of viral or bacterial infections and, consequently, the fever‐induced seizures and status epilepticus. Fever sensitivity is a hallmark of Dravet syndrome and is thought to result from impaired inhibitory control in thermosensitive brain circuits due to *SCN1A* pathogenic variants, which disrupt sodium channel function in GABAergic interneurons.^22^ Complementing this channelopathy‐based mechanism, emerging evidence points to a contributory role for inflammatory pathways.^23^, ^24^ For instance, monocytes from individuals with Dravet syndrome have shown altered cytokine responses to vaccines, suggesting a dysregulated innate immune response.^25^ These findings raise the possibility that IVIG could modulate fever‐associated neuronal hyperexcitability through both peripheral and central immunomodulatory effects. To our knowledge, no studies have systematically evaluated the immunological profile of individuals with Dravet syndrome. In our cohort, immunoglobulin levels were assessed before treatment initiation in 9 out of 14 individuals. No major immunoglobulin deficiencies were identified, although one patient presented with an isolated immunoglobin G1 subclass deficiency, with otherwise normal total immunoglobin G, immunoglobin A, and immunoglobin M levels. These findings suggest that classical humoral immunodeficiency is not a common feature in Dravet syndrome. However, routine immunological screening is rarely performed in this population, and the relevance of immune modulation remains to be better understood.

Evidence on the use of IVIG in epilepsy remains limited and challenging. A 2019 Cochrane review found no convincing evidence to support its efficacy in treating drug‐resistant epilepsy, underscoring the need for high‐quality randomized trials.^26^ However, IVIG has been increasingly recognized for its immunomodulatory role in specific epilepsy contexts. In particular, its use has been recommended as part of first‐line immunotherapy in new‐onset refractory status epilepticus and febrile infection‐related epilepsy syndrome, where immune dysregulation is suspected to contribute to pathogenesis. International consensus guidelines advocate for initiating immunotherapy, including IVIG, within 72 hours in these situations, particularly when no aetiology is identified.^27^ Although the mechanisms underlying Dravet syndrome are distinct, the fever sensitivity and frequent infection‐triggered seizures seen in early childhood may share some mechanistic overlap with these syndromes. These parallels support further exploration of IVIG in Dravet syndrome, particularly in individuals with frequent, fever‐associated status epilepticus. Our study contributes the largest series to date in this specific population, offering preliminary but structured insight into an empirically adopted therapeutic strategy.

This retrospective study has several limitations that must be acknowledged. The cohort included a small number of individuals, and the absence of a control group limits the interpretation of the observed effects, which should therefore be viewed as exploratory and descriptive rather than confirmatory. We chose the number of hospitalizations for status epilepticus as our primary outcome, as it was the most objective and consistently available measure in a retrospective setting. However, this does not capture the full spectrum of seizure burden. In addition, antiseizure medication changes occurred concomitantly with IVIG initiation in some cases, making it difficult to isolate the effect of IVIG alone. Additionally, the natural evolution of Dravet syndrome must be considered, as febrile status epilepticus tends to become less frequent with age. Given that IVIG was introduced during early childhood in most individuals, it is difficult to fully disentangle the effect of treatment from the expected age‐related decline in seizure severity. Seasonal variation may also have influenced outcomes, as several individuals initiated IVIG during winter months, followed by periods of lower viral circulation in spring or summer. Tolerability also raises concern as 6 out of 14 individuals experienced adverse events during IVIG infusion, mostly fever‐related seizures, and 6 out of 14 individuals required central venous access because of difficulties with venipuncture, carrying an inherent risk of complications. These practical constraints and safety concerns underscore the need for caution in considering IVIG as a preventive treatment in Dravet syndrome. Taken together, these findings support the rationale for a prospective, controlled clinical trial to better assess the efficacy, safety, and feasibility of IVIG in this population, and to determine whether it may represent a useful adjunctive option in selected cases.

### CONCLUSION

This study provides the first structured evaluation of IVIG for the prevention of prolonged febrile seizures in children with Dravet syndrome. While not a first‐line option, IVIG may represent a potential therapeutic avenue for selected individuals with frequent, fever‐triggered status epilepticus. These preliminary results support the need for prospective studies to better define their role.

## Data Availability

The data that support the findings of this study are available on request from the corresponding author. The data are not publicly available due to privacy or ethical restrictions.
